# Commentary: Prevalence and incidence of celiac disease in patients with rheumatoid arthritis: a case-control study based on the RECORD cohort

**DOI:** 10.3389/fmed.2025.1572552

**Published:** 2025-05-29

**Authors:** Eléonore Bettacchioli, Divi Cornec, Pauline Gardien, Lucille Quenehervé, Alain Saraux

**Affiliations:** ^1^LBAI, UMR1227, Inserm, University of Brest, Brest, France; ^2^Department of Immunology, CHU de Brest, Brest, France; ^3^Department of Rheumatology, CHU de Brest, Brest, France; ^4^Department of Gastroenterology, CHU de Brest, Brest, France

**Keywords:** celiac disease, rheumatoid arthritis, screening, serology, cost-benefit balance

## Introduction

We read with great interest the work of Sakellariou et al. which presented a well-structured and large-scale case-control study assessing the prevalence and incidence of celiac disease (CD) among patients with rheumatoid arthritis (RA) using administrative health data from the Lombardy region of Italy (1). They determined that the prevalence of CD was higher in RA as compared to healthy controls [171/70,061 = 0.24% (0.2%−0.3%) vs. 398/276,895 = 0.14% (0.1%−0.2%), *p* < 0.001], especially in females with RA (0.3% vs. 0.08%, *p* < 0.001) which aligns with prior epidemiological research (2–4). Their data also suggests a stable incidence over time. The authors propose that systematic screening for CD in RA patients, especially in high-risk subgroups such as female patients, could be warranted. However, we caution against adopting a widespread serological screening in RA patients through economic, epidemiological and clinical considerations.

## Economic considerations against systematic CD screening in RA

To assess the direct economic impact of a potential systematic CD screening in RA, we estimated the cost for male, female, and all RA patients within the cohort of patients studied by Sakellariou et al. (1). We notably calculated (i) the minimal cost of anti-transglutaminase IgA testing, considering reagent costs only, and (ii) the average cost of a large-scale screening strategy based on reimbursement rates provided by the French National Social Security System (15.08€/test). For reagent cost estimation, we used the IDS ISYS chemiluminescence test for anti-transglutaminase IgA, a widely used CD diagnostic assay, priced at 3.25€/test.

Our findings indicate that the total cost of a systematic screening strategy could range from 227,698€ to 1,056,520€ for all RA patients in the Sakellariou cohort (Table 1). Restricting screening to female RA patients, given their higher susceptibility to CD, would reduce the financial burden, with costs ranging from 160,758€ to 74,591€. The estimated mean cost per CD diagnosis varies from 1,079€ to 5,006€ for female patients and from 1,332€ to 6,178€ for all RA patients.

**Table 1:** Estimated cost of systematic celiac disease screening in RA patients in Sakellariou's cohort: reagent-based vs. French social security reimbursement.

	**Male RA patients**	**Female RA patients**	**All RA patients**
**Number of patients**			
Number of patients also diagnosed with CD, n (%)	22/20,595 (0,106%)	149/49,464 (0,301%)	171/70,061 (0,244%)
**Minimal cost**			
Minimal estimated cost for the screening of all patients (in €)	66,940	160,758	227,698
Mean estimated expense per diagnosis (in €)	3,042	1,079	1,332
**Average cost**			
Average estimated cost for the screening of all patients (in €)	310,603	745,917	1,056,520
Average estimated expense per diagnosis (in €)	14,119	5,006	6,178

Minimal estimated cost was determined based on reagent-cost for anti-transglutaminase IgA testing only. Average estimated cost was determined based on the French Social Security System reimbursement for anti-transglutaminase IgA testing.

These figures underscore the financial burden of systematic screening. Moreover, given that two-thirds of RA patients are women (5), limiting screening based on sex alone does not significantly reduce costs.

## Epidemiological considerations against systematic CD screening in RA

From an epidemiological perspective, the slightly increased risk of CD in RA does not necessarily justify routine screening in asymptomatic individuals. Current guidelines recommend screening in high-risk groups like patients with type 1 diabetes, where the CD prevalence is significantly higher (6, 7). Both type 1 diabetes and CD typically manifest in early life, whereas RA has a later onset, suggesting that most cases of CD in RA patients should have already been diagnosed. Moreover, given that CD prevalence in RA does not show a steady increase over time (1), a symptom-based screening approach remains preferable over population-wide testing.

Interestingly, Sakellariou et al. observed a higher risk of CD in younger RA patients but not in those over 50 years old. One possible explanation is the influence of media-driven self-diagnosis of CD among younger individuals. Many young patients who read that gluten may contribute to arthritis might experiment with a gluten-free diet and self-diagnose as having CD (8). Because of the administrative nature of Sakellariou et al.'s data without serological status or biopsy results, a significant limitation arises in confirming true CD diagnoses in RA patients.

## Real-life challenges regarding a potential systematic CD screening in RA

To evaluate the feasibility of systematic CD screening in RA in real-life settings, we conducted a study involving over 1,400 patients with early inflammatory rheumatisms including 700 RA (9). This cohort consisted of significantly younger individuals at disease onset, making it an ideal candidate for assessing the potential effectiveness of systematic screening. We employed serological screening followed by medical confirmation to establish definitive CD diagnoses. Among the eight positive tests, only one CD case was confirmed, co-occurring with Sjögren's Disease, which may have been the primary predisposing factor rather than RA itself. Thus, systematic CD screening entailed substantial financial costs with very limited diagnostic yield, reinforcing our earlier economic analysis.

It should be noted that current guidelines recommend confirming positive serology with duodenal biopsies (10), meaning that in our cohort, 7 RA patients required endoscopy to confirm or refute the diagnosis. A recent meta-analysis (11) demonstrated the excellent specificity of anti-transglutaminase IgA levels ≥10 times the upper limit of normal for diagnosing CD, but most patients have lower levels, requiring additional endoscopic evaluation. These patients have to undergo potentially unnecessary endoscopy, face prolonged uncertainty, and bear the psychological burden of envisioning the diagnosis of a chronic disease requiring a restrictive gluten-free diet.

## Conclusion

The findings of Sakellariou et al. are in line with previous literature indicating a higher prevalence of CD in autoimmune diseases, including RA (1–4), raising the question of whether systematic screening for CD in RA is beneficial. However, our analysis suggests that such an approach carries significant economic, clinical, and epidemiological limitations.

The financial burden remains high, even when restricted to high-risk groups, with a low diagnostic yield. In addition, potential long-term follow-up costs, such as repeated serological testing, monitoring, or management of false positives (not included in our cost analysis), could further increase the overall financial impact of systematic screening strategies. Epidemiological data indicate that most CD cases in RA patients should have already been diagnosed due to differing age onsets, while self-diagnosis trends complicate prevalence assessments. Finally, real-world considerations further emphasize the challenges of systematic screening, with low detection rates, unnecessary biopsies, and heightened patient anxiety.

Future research may help refine risk stratification criteria for CD screening in RA by incorporating epidemiological data such as age and sex, multiple clinical features, coexisting autoimmune diseases, and genetic markers such as HLA-DQ2 or HLA-DQ8 to identify a small population at very high risk and candidate for systematic screening (12). Moreover, multi-center studies could provide more robust information to support evidence-based screening recommendations. Finally, further cost-effectiveness analyses would be crucial before implementing widespread screening recommendations. Until then, a targeted approach, prioritizing patients with evocative symptoms of CD or associated autoimmune conditions, remains the most pragmatic strategy (7, 12).
